# Comparison of parallel infill sampling criteria based on Kriging surrogate model

**DOI:** 10.1038/s41598-021-04553-5

**Published:** 2022-01-13

**Authors:** Cong Chen, Jiaxin Liu, Pingfei Xu

**Affiliations:** Wuhan Second Ship Design and Research Institute, The Third Research Laboratory, Wuhan, 430064 China

**Keywords:** Engineering, Aerospace engineering, Mechanical engineering

## Abstract

One of the key issues that affect the optimization effect of the efficient global optimization (EGO) algorithm is to determine the infill sampling criterion. Therefore, this paper compares the common efficient parallel infill sampling criterion. In addition, the pseudo-expected improvement (EI) criterion is introduced to minimizing the predicted (MP) criterion and the probability of improvement (PI) criterion, which helps to improve the problem of MP criterion that is easy to fall into local optimum. An adaptive distance function is proposed, which is used to avoid the concentration problem of update points and also improves the global search ability of the infill sampling criterion. Seven test problems were used to evaluate these criteria to verify the effectiveness of these methods. The results show that the pseudo method is also applicable to PI and MP criteria. The DMP and PEI criteria are the most efficient and robust. The actual engineering optimization problems can more directly show the effects of these methods. So these criteria are applied to the inverse design of RAE2822 airfoil. The results show the criterion including the MP has higher optimization efficiency.

## Introduction

Optimization problems are often involved in engineering design^1^. The objective function values and constraints are mainly obtained by numerical simulation or experiment. Although the population-based algorithm (genetic algorithm, particle swarm algorithm, etc.) can get a reliable optimal solution. The difficulty is that they tend to require hundreds of function evaluations to find good parameter sets.

The surrogate model can replace the expensive calculation. Among many alternative models, Kriging is one of the most popular models^2–4^. Its main advantage is not only to provide the numerical response of the sample but also to provide the estimation error, which provides a way for improving the accuracy of the surrogate model.

The efficient global optimization (EGO) algorithm^5^ is one of the most frequently studied and widely used optimization algorithms for solving computationally expensive optimization problems. The surrogate model constructed with initial samples is often not directly used for optimization. Because unreasonable initial sample will make the model not have global accuracy. Therefore, it is necessary to add the sample points to improve the initial model. Conventional single-point infill sampling criteria include EI, MP, PI, MES (maximizing the predicted error), and LCB (lower confidence bounding) criteria^6,7^. Parallel infill sampling criteria can be divided into three categories based on how many optimization methods and how many criteria are used to generate updated points at each cycle. The first is to generate multiple sample points in each cycle using one criterion. Ginsbourger et al.^8^ derived the q-point expected improvement criterion. However, when the dimension of the test problem is greater than two, there is no analytical solution. It needs to be solved by the Monte-Carlo method. Soberster et al.^9^ proposed a method to select multiple local maximum points of EI. Feng et al.^10^ divided the EI into two parts and obtained the Pareto solutions of the two parts by using the multi-objective optimization algorithm. The same approach is applied to the weight EI and generalized EI criteria^11,12^. Li et al.^13^ extended the GEGO method with the improved constant liar (CL) strategy The main disadvantage of the above methods is that the number of candidate points is uncertain. Accordingly, many methods have been produced to control the number of candidate points.

The second is to perform multiple optimizations in each cycle using one criterion. The most typical method is Kriging Believer (KB) developed by Ginsbourger^8^. First, the first candidate point is selected by the standard EI criterion. A ‘fake’ value is selected to update the sample data and the Kriging model. Then the updated model is used to find the next maximum EI point. The ‘fake’ value is mainly using the Kriging prediction value or a constant value. The introduction of “fake” values can reduce the time required to evaluate true function values. The principle is that the EI value near the current point will be reduced by adding a ‘fake’ value. Then the next maximum EI point can be found by optimization. Viana et al.^14^ directly introduce a fixed distance to reduce the EI value near the candidate solution. Similarly, Zhan et al.^15^ introduced an influence function to update points and the criterion is called pseudo-expected improvement (PEI) .

The third is to use multiple initial sample criteria^16^ or the surrogate models^17^. The drawbacks are the number of candidate points is limited and it is easy to generate duplicate points. Moreover, multiple criteria can be selected for multi-objective optimization^18^. The problem of selecting points is followed.

It can be found the second criterion is simple to implement and can control the number of candidate points. The design idea of the pseudo-EI criterion is to use the influence function to approximate the influence of the update point on the EI function when the Kriging model is updated. Therefore, the second updated point can be selected without updating the model. This method is easier to implement and can be directly introduced into other criteria. At the same time, it can improve the problem that other methods tend to fall into local optimum. This paper introduces the idea of pseudo-EI criterion into PI criterion and MP criterion. Because the influence function can reflect the influence of the update point. Therefore, the author improves the traditional fixed distance function by setting a threshold for the influence function. The author introduces distance functions into the PI, MP, and EI criteria.

In this paper, first, we take a one-dimensional function as an example to illustrate whether the multiple update points obtained by parallel methods in one iteration can achieve the effect of multiple iterations with the traditional single infill sampling criteria. It can also show the similarities and differences of various parallel infill sampling criteria more intuitively.

The second part selects common low-dimensional and high-dimensional test functions to verify the effectiveness of the parallel infill sampling criteria proposed in this paper and compares the difference in optimization efficiency and robustness with traditional parallel criteria. It can provide a reference for other people to choose the parallel criteria.

Furthermore, in the third part, several parallel infill sampling criteria are applied to the actual inverse design of two-dimensional airfoil. Comparing the difference in the optimal speed and optimal value of different criteria and updating points, which is beneficial to provide a reference for airfoil optimization.

## The efficient global optimization algorithm

### Kriging model

Kriging model regards the target function as a combination of a regression model and a stochastic process. The regression model usually uses a constant. The initial samples are $S = \left\{ {x^{1} ,x^{2} , \ldots ,x^{{\text{n}}} } \right\}^{T} \in {\mathbb{R}}^{{n*n_{\dim } }}$. The corresponding function values are $y = \left\{ {y^{1} \left( x \right),y^{2} \left( x \right), \ldots ,y^{{\text{n}}} \left( x \right)} \right\}^{T} \in {\mathbb{R}}^{{n*n_{\dim } }}$

1 $$y\left( x \right) = \mu + z\left( x \right)$$

where $\mu$ is a constant, $z\left( x \right)$ is the stochastic process assumed to have mean zero and covariance

2 $${\text{cov}}\left( {z\left( x \right),z\left( v \right)} \right) = \sigma^{2} \Re \left( {x,v,\theta } \right)$$

between $z\left( x \right)$ and $z\left( v \right)$, where $\sigma^{2}$ is the process variance of the response and $\Re \left( {x,v,\theta } \right)$ is the correlation model with parameters $\theta$. The correlation model is a function of the distance between the sample points. When the distance between the two points is small, the value of the correlation function is near one. As the distance increases, the value tends to zero.

For an n-dimensional problem, the correlation function $\Re \left( {x,v,\theta } \right)$ that represents the correlation between point *x*(*j*) and point *v*(*j*) is shown by

3 $$\Re \left( {x_{j} ,v_{j} ,\theta } \right) = \mathop \prod \limits_{k = 1}^{n} \Re (\theta_{k} ,x_{k}^{j} - v_{k}^{j} )$$

Based on the unbiased estimation ${\text{E}}\left[ {\hat{y}\left( x \right) - y\left( x \right)} \right] = 0$, the corresponding maximum likelihood estimate of the variance is:

4 $$\sigma^{2} \left( x \right) = \frac{1}{n}(Y - {\mathbf{1}}\mu ){\varvec{R}}^{ - 1} \left( {Y - {\mathbf{1}}\mu } \right)$$

A detailed description can be found in^2^. Kriging predictor and the mean squared error can be obtained as follows.

5 $$\hat{y}\left( x \right) = \mu + {\varvec{r}}\left( x \right)^{T} {\varvec{R}}^{ - 1} \left( {Y - {\mathbf{1}}\mu } \right)$$

6 $$\hat{s}\left( x \right) = \hat{\sigma }^{2} \left( {{\mathbf{1}} + {\varvec{r}}^{T} {\varvec{R}}^{ - 1} {\varvec{r}} + \left( {{\mathbf{1}}^{T} {\varvec{R}}^{ - 1} {\varvec{r}} - {\mathbf{1}}} \right)^{T} \left( {{\mathbf{1}}^{T} {\varvec{R}}^{T} {\mathbf{1}}} \right)\left( {{\mathbf{1}}^{T} {\varvec{R}}^{T} {\varvec{r}} - {\mathbf{1}}} \right)} \right)$$

In the equations, ***R*** is a matrix with entry $R_{ij} \left( x \right) = \Re \left( {x_{i} ,x_{j} ,\theta } \right)$, ***r*** is a $n_{\dim }$-dimensional vector with entry $r_{i} \left( x \right) = \Re \left( {x,x_{i} ,\theta } \right)$, and **1** is $n_{\dim }$-dimensional vector of ones.

### Minimizing the prediction criterion

The premise of applying this criterion is that the surrogate model is accurate. The criterion directly searches for the minimum objective based on the surrogate models. But it is easy to fall into the local optimum trap. In this paper, to find the maximum value of the criterion uniformly, the MP criterion is negative.

7 $$MP = \min \left( {\hat{y}\left( x \right)} \right)$$

### Expected improvement criterion

The expected improvement is defined as the mathematical expectation of the improvement value at a predicted point^5^. The improvement can be defined as $I\left( x \right) = \max \left( {y_{min} - y\left( x \right),0} \right)$. Then the expected improvement is given by

8 $$EI\left( x \right) = \left( {y_{min} - \hat{y}\left( x \right)} \right)\Phi \left( {\frac{{y_{min} - \hat{y}\left( x \right)}}{{\hat{s}\left( x \right)}}} \right) + \hat{s}\phi \left( {\frac{{y_{min} - \hat{y}\left( x \right)}}{{\hat{s}\left( x \right)}}} \right)$$

where $\Phi \left( x \right)$ and $\phi \left( x \right)$ are the cumulative distribution and probability density function of the standard normal distribution, respectively.

It can be seen from the formula of EI that it considers both the prediction value and the prediction error of the Kriging model. Thus it gives a balance between local search and global search.

### Probability of improvement criterion

The probability of improvement is defined as the probability that the predicted point value is less than the current true optimal value^17^. In general, we want to achieve a smaller value $T$ than the current true optimal value. The value of $T$ is as follows:

9 $$T = y_{\min } - \kappa \left| {y_{\min } } \right|$$

where $\kappa$ is the improvement factor. The $\kappa$ is set to 0.1^6^.

The probability of improvement is described below:

10 $$PI\left( x \right) = \Phi \left( {\frac{{T - \hat{y}\left( x \right)}}{{\hat{s}\left( x \right)}}} \right)$$

### Pseudo expected improvement criterion

The principle is to reduce the value near the updated points by introducing influence functions. The influence function is shown in (11)^15^.

11 $$IF\left( {x,x^{add} } \right) = 1 - R\left( {x,x^{add} } \right)$$

The first updated point is identified by maximizing the standard EI function. As the optimization process goes on, the ${\varvec{q}}$th updated point is identified as:

12 $$\begin{aligned} x^{{\left( {n + q} \right)}} & = \max \left( {PEI\left( {x,x^{{\left( {n + 1} \right)}} , \ldots ,x^{{\left( {n + q - 1} \right)}} } \right)} \right) \\ & = \max \left( {EI\left( x \right)*\prod\limits_{i = 1}^{q - 1} {\left( {IF\left( {x,x^{{\left( {n + q - 1} \right)}} } \right)} \right)} } \right) \\ \end{aligned}$$

The detailed adding process can be seen at the end of this section. Similarly, we use the same steps to apply the pseudo method to MP and PI criteria.

13 $$\begin{aligned} x^{{\left( {n + q} \right)}} & = \max \left( {PMP\left( {x,x^{{\left( {n + 1} \right)}} , \ldots ,x^{{\left( {n + q - 1} \right)}} } \right)} \right) \\ & = \max \left( {MP\left( x \right)*\prod\limits_{i = 1}^{q - 1} {\left( {IF\left( {x,x^{{\left( {n + q - 1} \right)}} } \right)} \right)} } \right) \\ \end{aligned}$$

14 $$\begin{aligned} x^{{\left( {n + q} \right)}} & = \max \left( {PPI\left( {x,x^{{\left( {n + 1} \right)}} , \ldots ,x^{{\left( {n + q - 1} \right)}} } \right)} \right) \\ & = \max \left( {PI\left( x \right)*\prod\limits_{i = 1}^{q - 1} {\left( {IF\left( {x,x^{{\left( {n + q - 1} \right)}} } \right)} \right)} } \right) \\ \end{aligned}$$

### Distance function

Viana et al.^17^ introduced a fixed distance to constrain updated points. The distance of each update point in each loop is greater than the given value. The distance is defined as $0.1*\sqrt {n_{\dim } }$. $n_{\dim }$ is the number of dimensions of the test problem. The parallel minimizing the prediction criterion with a fixed distance to constrain is referred to as the MMP criterion. The parallel minimizing the prediction criterion with a fixed distance to constrain updated points is referred to as the MMP criterion. The parallel probability of improvement with a fixed distance to constrain updated points is referred to as the MPI criterion. The parallel expected improvement with a fixed distance to constrain is referred to as the MEI criterion. The range of influence of the real updated point is not the same as the given distance. So it is not reasonable to use a fixed distance directly. An adaptive distance constraint is proposed based on the PEI. The method also uses correlation functions. When the value of the correlation function is less than the threshold $\delta \in (0,1)$, the value of the distance function is 1. Otherwise, it is 0. The distance function not only avoids the concentration of the candidate solution but also improves the global searching ability. Because the hyper-parameters are anisotropic, so the distance function is also anisotropic in all directions.

15 $$DIS\left( {x,x^{add} } \right) = \left\{ {\begin{array}{*{20}l} 1 \hfill & {R\left( {x,x^{add} } \right) < \delta } \hfill \\ 0 \hfill & {R\left( {x,x^{add} } \right) > \delta } \hfill \\ \end{array} } \right.$$

Applying this method can refer to Viana’s^17^ or Zhan’s^15^. In this paper, the method of Zhan^15^ is taken as an example. The $q$th updated point is identified as:

16 $$\begin{aligned} x^{{\left( {n + q} \right)}} & = \max \left( {DEI\left( {x,x^{{\left( {n + 1} \right)}} , \ldots ,x^{{\left( {n + q - 1} \right)}} } \right)} \right) \\ & = \max \left( {EI\left( x \right)*\prod\limits_{i = 1}^{q - 1} {\left( {DIS\left( {x,x^{{\left( {n + q - 1} \right)}} } \right)} \right)} } \right) \\ \end{aligned}$$

17 $$\begin{aligned} x^{{\left( {n + q} \right)}} & = \max \left( {DMP\left( {x,x^{{\left( {n + 1} \right)}} , \ldots ,x^{{\left( {n + q - 1} \right)}} } \right)} \right) \\ & = \max \left( {MP\left( x \right)*\prod\limits_{i = 1}^{q - 1} {\left( {DIS\left( {x,x^{{\left( {n + q - 1} \right)}} } \right)} \right)} } \right) \\ \end{aligned}$$

18 $$\begin{aligned} x^{{\left( {n + q} \right)}} & = \max \left( {DPI\left( {x,x^{{\left( {n + 1} \right)}} , \ldots ,x^{{\left( {n + q - 1} \right)}} } \right)} \right) \\ & = \max \left( {PI\left( x \right)*\prod\limits_{i = 1}^{q - 1} {\left( {DIS\left( {x,x^{{\left( {n + q - 1} \right)}} } \right)} \right)} } \right) \\ \end{aligned}$$

**Figure 1:**
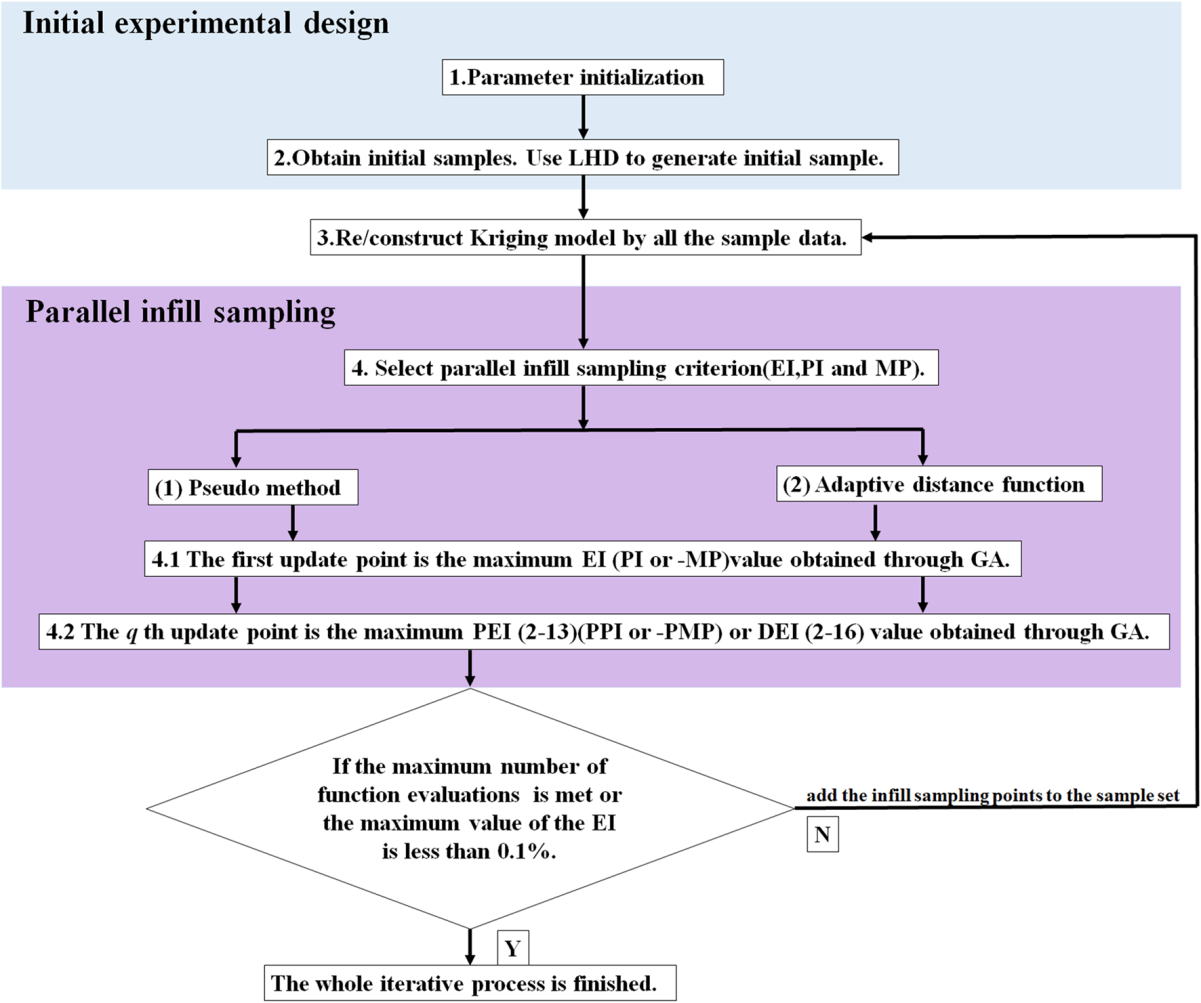
Flowchart of the parallel updated points.

Figure 1 shows the flowchart of the parallel updated points. The specific optimization process is as follows.

Step 1: Parameter initialization. The parameters, including design domain, boundary constraints, relative parameters of the Kriging model, number of updating points, and convergence condition are initialized.

Step 2: Obtain initial samples by LHD.

Step 3: Build a Kriging model based on the current design samples.

Step 4: Take the expected improvement (EI) criterion as an example to illustrate the process of parallel adding points.

1. Pseudo method.
  - Identify the first update point by looking for the maximum EI function value using GA.
  - An approximation of the updated EI function is calculated by multiplying the initial EI function by the influence function of the point $x^{{\left( {n + 1} \right)}}$:
    19 $$PEI\left( {x,x^{{\left( {n + 1} \right)}} } \right) = EI\left( x \right)*\left( {IF\left( {x,x^{{\left( {n + 1} \right)}} } \right)} \right)$$
  - The second updating point is produced by maximizing the approximated updated EI function:
    20 $$x^{{\left( {n + 1} \right)}} = \max \left( {PEI\left( {x,x^{{\left( {n + 1} \right)}} } \right)} \right)$$
  - As the optimization process goes on, the ${\varvec{q}}$th updated point is identified as (13):
2. Fix distance function method.
  - Identify the first update point by looking for the maximum EI function value.
    21 $$D\left( {x,x^{add} } \right) = \left\{ {\begin{array}{*{20}l} 1 \hfill & {{\text{if}}\;Euclidean\;distance > 0.1*\sqrt {n_{\dim } } } \hfill \\ { - 1} \hfill & {else\;Euclidean\;distance > 0.1*\sqrt {n_{\dim } } } \hfill \\ \end{array} } \right.$$
  - The second updating point is identified as:
    22 $$x^{{\left( {n + 1} \right)}} = \max \left( {EI\left( x \right)*\left( {D\left( {x,x^{{\left( {n + 1} \right)}} } \right)} \right)} \right)$$
  - As the optimization process goes on, the $q$th updated point is identified as:
    23 $$x^{{\left( {n + q} \right)}} = \max \left( {EI\left( x \right)*min\left( {D\left( {x,x^{{\left( {n + 1} \right)}} } \right),D\left( {x,x^{{\left( {n + 2} \right)}} } \right), \ldots ,D\left( {x,x^{{\left( {n + q} \right)}} } \right)} \right)} \right)$$
3. Adaptive distance function.
  - Identify the first update point by looking for the maximum EI function value.
  - The second updating point is produced by maximizing the approximated updated EI function:
    24 $$x^{{\left( {n + 1} \right)}} = \max \left( {EI\left( x \right)*\left( {DIS\left( {x,x^{{\left( {n + 1} \right)}} } \right)} \right)} \right)$$
  - As the optimization process goes on, the $q$th updated point is identified as (16):

Step 5: Convergence conditions. If the maximum number of function evaluations is met or the maximum value of the EI is less than 0.1%, the whole loop is terminated. If it is not met, add the sampling points after the choice to the sample set, and back to Step 3.

To make the progress of parallel adding points more intuitive, the author uses a one-dimensional function in “Demonstrative example” to compare the process of single and parallel adding points.

## Numerical experiments

### Demonstrative example

**Figure 2:**
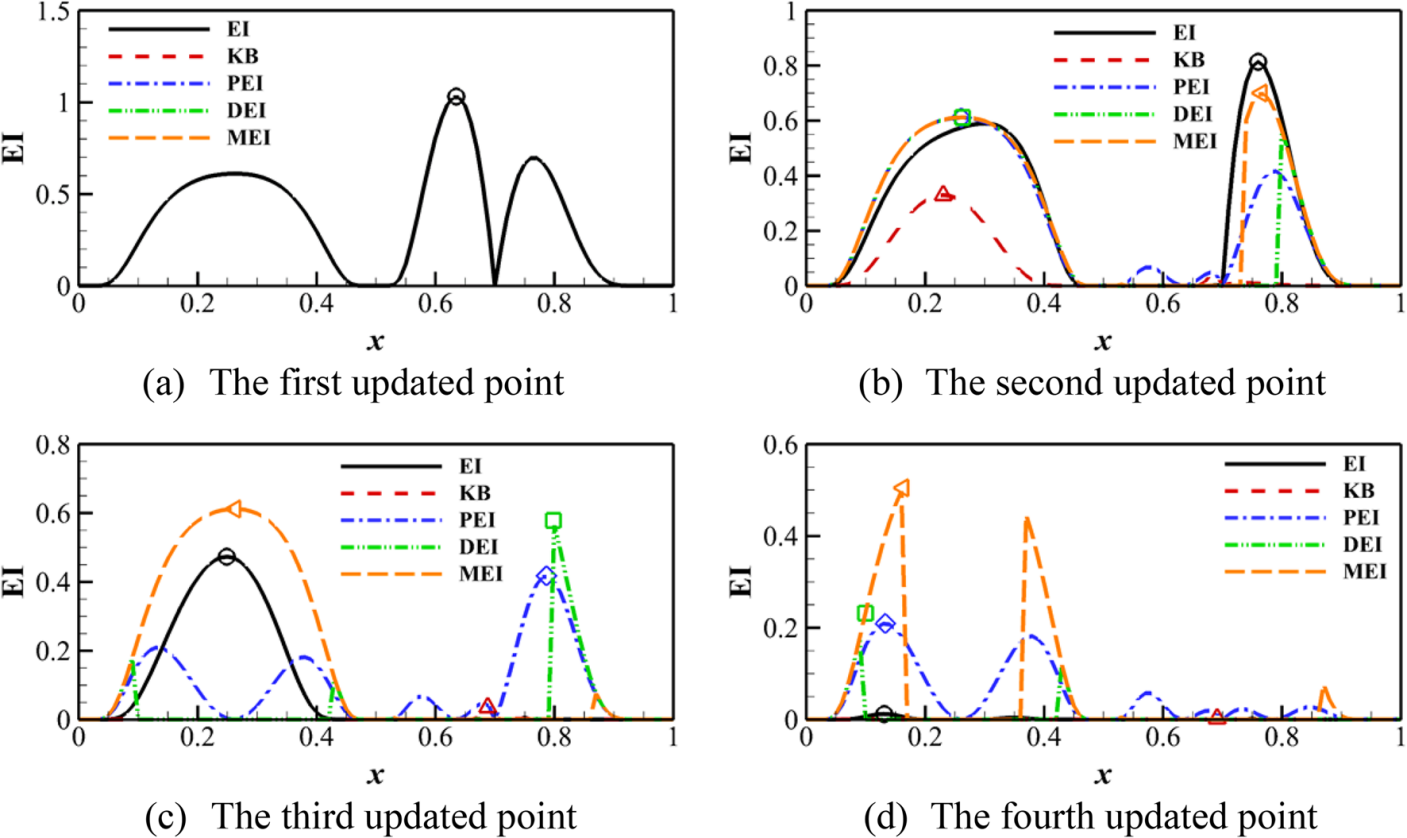
KB, PEI, DEI, and MEI algorithm on Forrester function compared with the standard EI algorithm.

**Figure 3:**
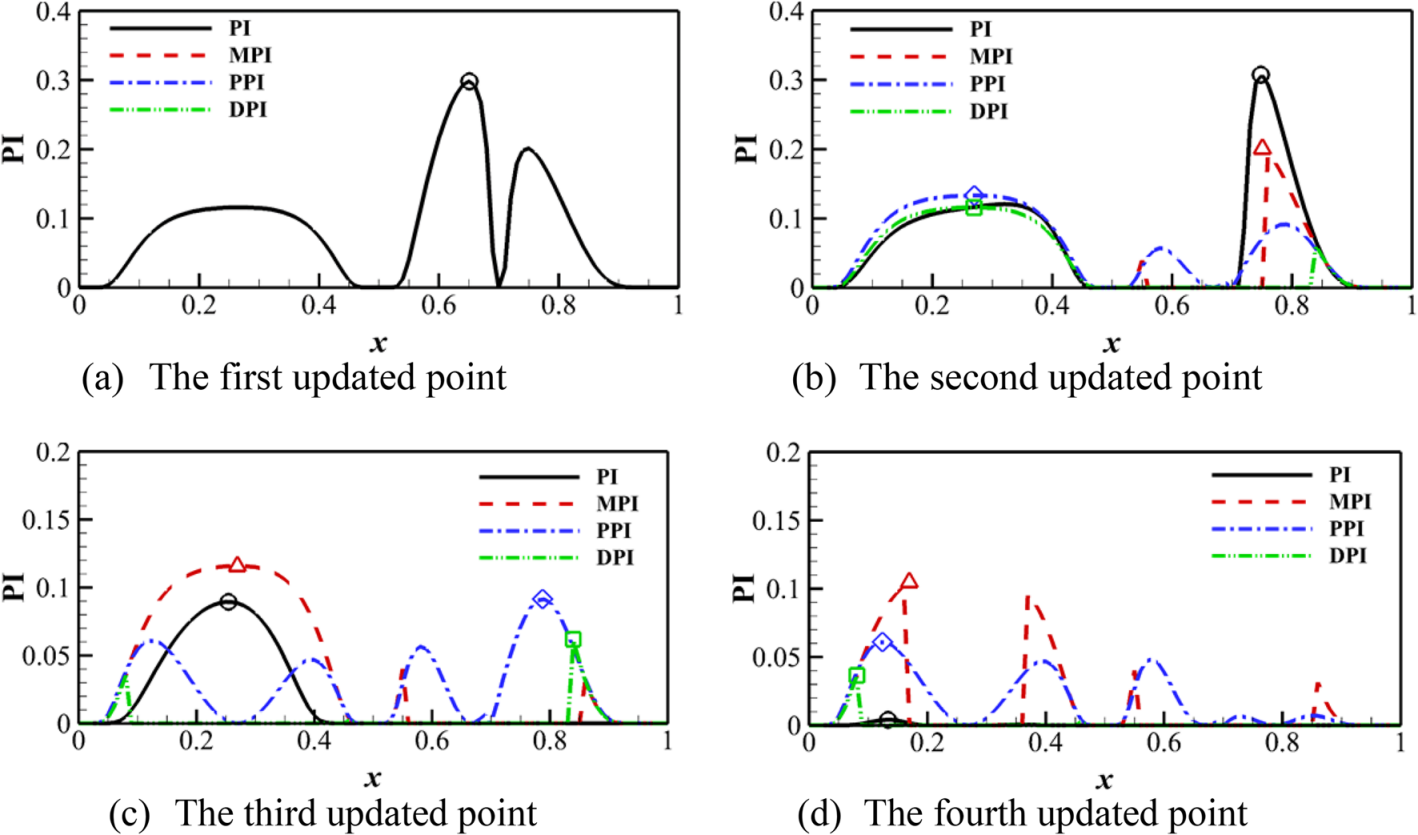
MPI, PPI and DPI algorithm on Forrester function compared with the standard EI algorithm.

The Forrester function^19^ $\left( {y = \left( {6x - 1} \right)^{2} \sin \left( {12x - 4} \right),x \in \left[ {0,1} \right]} \right)$ is used to demonstrate the process of updating points. The initial sampling points are $X = \left[ {0,0.5,0.7,1} \right]$. Four updating points are given in each cycle or iteration for the parallel criterion. Four cycles are demonstrated for the standard single-point criterion. Figure 2 shows the algorithm based on the EI criterion. The updated point position of the PEI, DEI, and MEI criteria is close to the standard EI criterion. The difference is the order of adding the point. For the KB criterion, the decay of the EI value is rapid because of the change of the hyper-parameter after introducing the ‘fake’ point. The other three only construct the Kriging model once, so the EI function is real. Therefore, it can be found that the parallel process essentially looks for other local optimal values. DEI and MEI criteria are similar in the first cycle, but the distance function will change with the update of the Kriging model for DEI. Figure 3 shows the algorithm based on the PI criterion. The updated points of the PI criterion are almost consistent with the EI criterion, which shows that the PI criterion is also an effective infilling samples method. And the updated points of MPI, PPI, and DPI criteria are also similar to the PEI, DEI, and MEI criteria. Therefore, the parallel probability of improvement (PI) criterion can obtain effective updated points.

**Figure 4:**
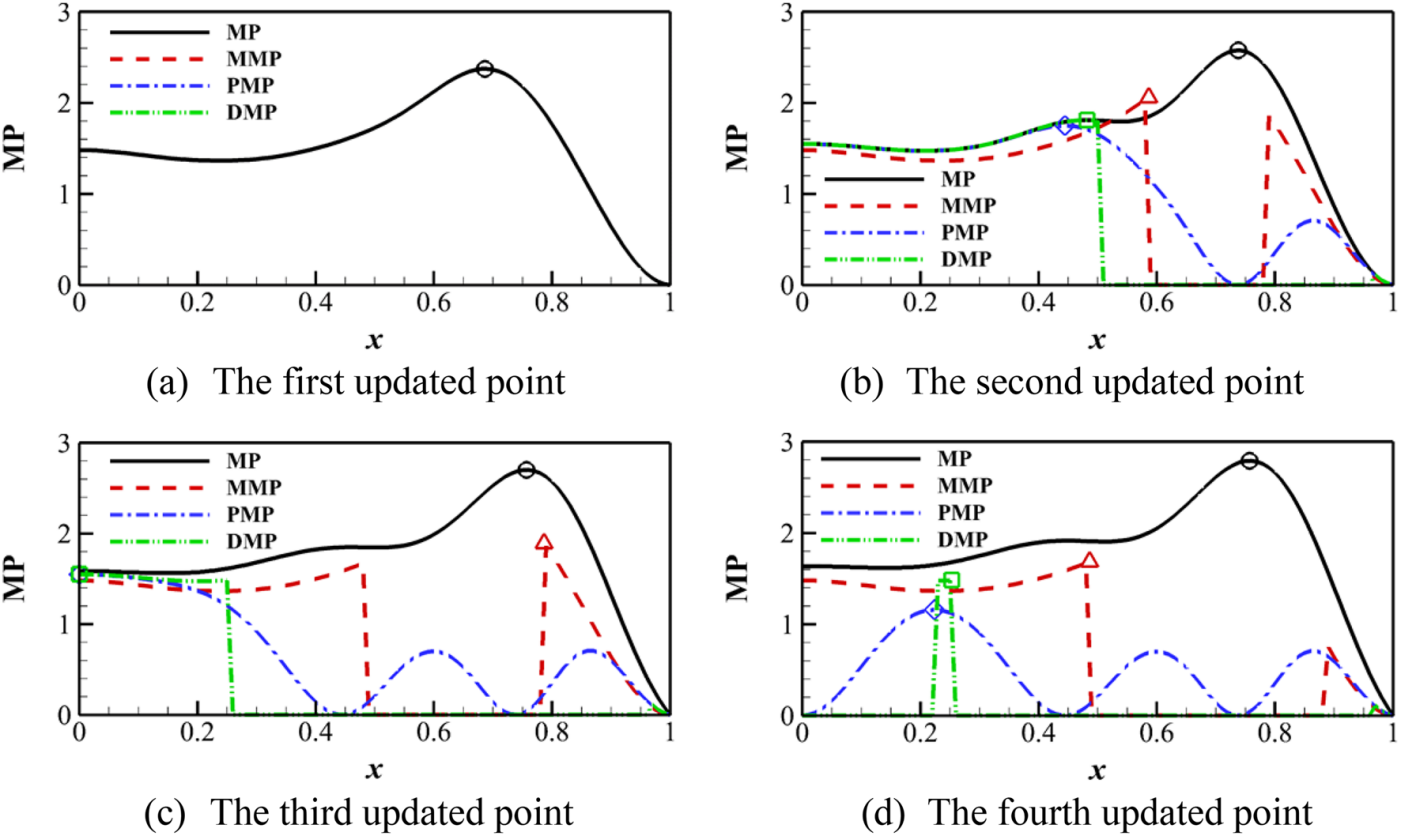
MMP, PMP, DMP algorithm on Forrester function compared with the standard EI.

Figure 4 shows the algorithm based on the MP criterion. The standard MP criterion has the poor global searching ability. Therefore, the candidate points are concentrated near the current optimal value. Although the optimal solution can be found when the surrogate model is accurate, it is not conducive to improving the prediction accuracy of the model. After the parallel criterion is introduced, the points near the candidate points cannot be added so that the search expands outward. It is conducive to finding local optimal values and improving the global searching ability.

Through the demonstration of a one-dimensional function, the parallel criteria proposed in this paper can effectively find update points after being introduced into the PI criterion. At the same time, the MP criterion can be prevented from falling into the local optimum.

### Test problems

Seven test problems are used for this experiment and described below.

Branin function with $n_{\dim } = 2$:

25 $$f = \left( {x_{2} - \frac{5}{4\pi }x_{1} - 6} \right)^{2} + 10\left( {1 - \frac{1}{8\pi }} \right)\cos \left( {x_{1} } \right) + 10,x_{1} \in \left[ { - 5,10} \right],x_{2} \in \left[ {0,15} \right]$$

Sasena function with $n_{\dim } = 2$:

26 $$\begin{aligned} & f = 2 + 0.01\left( {x_{2} - x_{1}^{2} } \right)^{2} + \left( {1 - x_{1} } \right)^{2} 2\left( {2 - x_{1} } \right) + 7\sin \left( {0.5x_{1} } \right)\sin \left( {0.7x_{1} x_{2} } \right) \\ & \quad x_{1,2} \in \left[ {0,5} \right] \\ \end{aligned}$$

Hartman with $n_{\dim } = 3$ and $n_{\dim } = 6$:

27 $$\begin{aligned} & f = - \sum\limits_{i = 1}^{4} {c_{i} } \exp \left[ { - \sum\limits_{j = 1}^{n} {\alpha_{ij} \left( {x_{j} - p_{ij} } \right)^{2} } } \right]x_{i} \in \left[ {0,1} \right],i = 1,2, \ldots ,n \\ & c = \left[ {1,1.2,3,3.2} \right],A\left( {n = 3} \right) = \left[ {\begin{array}{*{20}c} 3 & {10} & {30} \\ {0.1} & {10} & {35} \\ 3 & {10} & {30} \\ {0.1} & {10} & {35} \\ \end{array} } \right] \\ & A\left( {n = 3} \right) = \left( {\begin{array}{*{20}c} {10} & 3 & {17} & {3.5} & {1.7} & 8 \\ {0.05} & {10} & {17} & {0.1} & {8.0} & {14} \\ 3 & {3.5} & {1.7} & {10} & {17} & 8 \\ {17} & 8 & {0.05} & {10} & {0.1} & {14} \\ \end{array} } \right) \\ \end{aligned}$$

$$\begin{aligned} & P\left( {n = 3} \right) = \left[ {\begin{array}{*{20}c} {0.3689} & {0.1170} & {0.2673} \\ {0.4699} & {0.4378} & {0.7470} \\ {0.1091} & {0.8732} & {0.5547} \\ {0.03815} & {0.5743} & {0.8828} \\ \end{array} } \right] \\ & P\left( {n = 3} \right) = \left( {\begin{array}{*{20}c} {0.1312} & {0.1696} & {0.5569} & {0.0124} & {0.8283} & {0.5886} \\ {0.2329} & {0.4135} & {0.8307} & {0.3736} & {0.1004} & {0.9991} \\ {0.2348} & {0.1451} & {0.3522} & {0.2883} & {0.3047} & {0.6650} \\ {0.4047} & {0.8828} & {0.8732} & {0.5743} & {0.1091} & {0.0381} \\ \end{array} } \right) \\ \end{aligned}$$

StyblinskiTang7 with $$n_{\dim } = 7$$:

28 $$f = \frac{1}{2}\sum\limits_{i = 1}^{7} {\left( {x_{i}^{4} - 16x_{i}^{2} + 5x_{i} } \right)} ,x_{i} \in \left[ { - 5,5} \right],i = 1,2,...,7$$

Rosenbrock10 with $$n_{\dim } = 10$$:

29 $$f = - \sum\limits_{i = 1}^{9} {\left[ {100\left( {x_{i + 1}^{{}} - x_{i}^{2} } \right)^{2} + \left( {x_{i} - 1} \right)^{2} } \right]} ,x_{i} \in \left[ { - 2,2} \right],i = 1,2,...,10$$

Rastrigin12 with $$n_{\dim } = 12$$:

30 $$f = 120 + \sum\limits_{i = 1}^{12} {\left[ {x_{i}^{2} - 10\cos \left( {2\pi x_{i} } \right)} \right]} ,x_{i} \in \left[ { - 1,3} \right],i = 1,2,...,12$$

### Initial parameter setting

The initial sample uses the Latin hypercube design (LHD) function provided by MATLAB. Kriging model is built using the DACE toolbox^20^. The infill sampling criterion is optimized by the genetic algorithm provided by MATLAB. The settings are as follows.

- The number of initial points: $2 \times \left( {n_{\dim } + 1} \right)$
- Regression function: regpoly0.
- Correlation function: corrgauss.
- Initial hyperparameters:1
- Population size: 100
- Maximum iterations of GA: 100.

### Test results

All parallel criteria are run to produce 2, 4, 6, 8, and 10 updated points in each cycle or iteration. When the relative error of the optimal value is less than 0.01, we believe that convergence has been achieved. For the first four test problems, the maximum number of cycles is 100. The number of cycles is counted when optimization is done. The distance function threshold is set to 0.1. For the 5th to 7th test problems, because it is difficult to reach the optimal value for the high dimensional problems using 100 iterations. The maximum number of cycles is set to 20, 40, 60, 80, corresponding to the process of adding 2, 4, 6, 8, and 10 update points. The optimal value is counted, when the number of cycles reaches. The threshold value of the distance function is set to 0.5.

**Figure 5:**
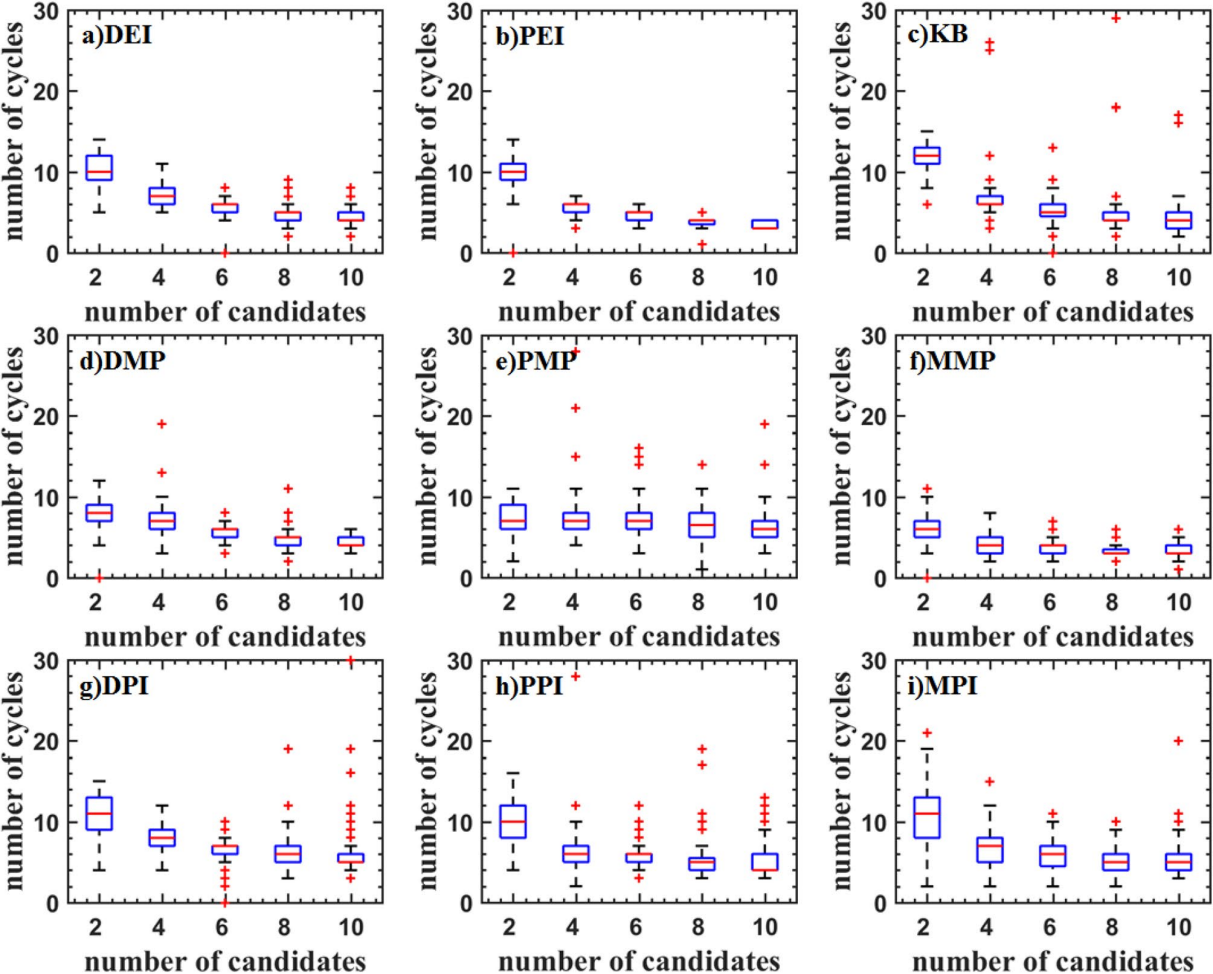
Test problems: Branin function.

**Figure 6:**
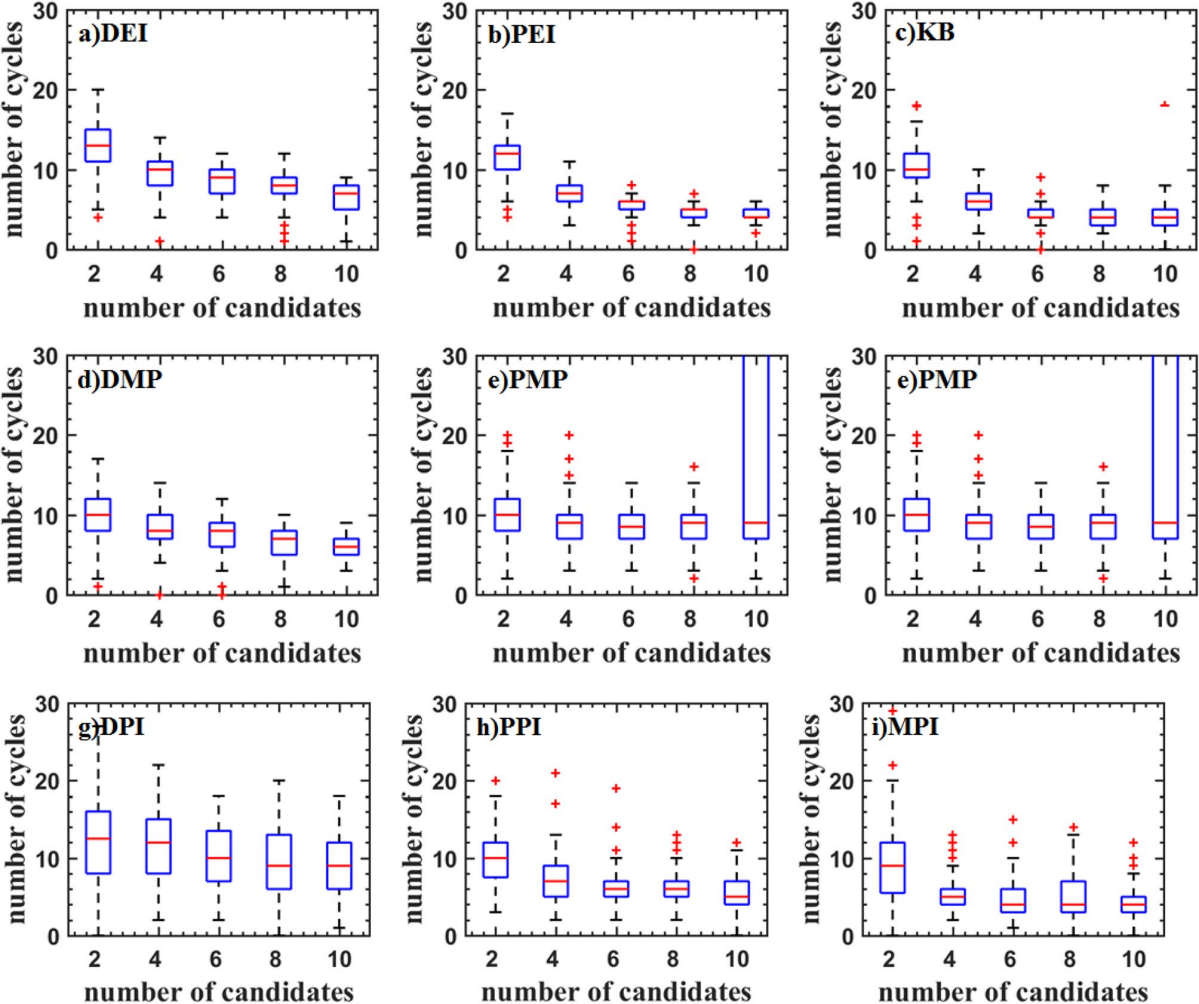
Test problems: Sasena function.

**Figure 7:**
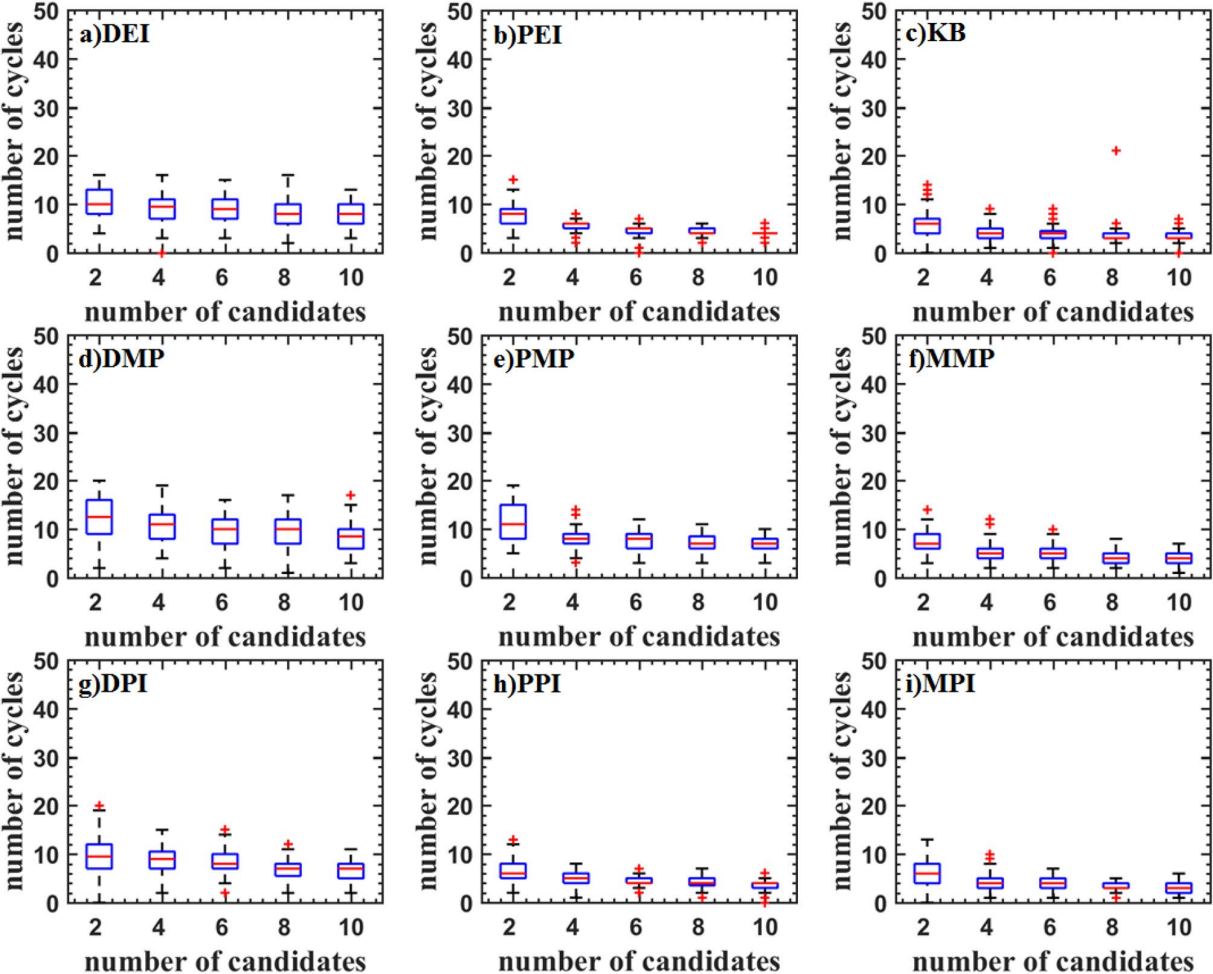
Test problems: Hartman3 function.

Figures 5, 6, and 7 show the boxplot of two and three-dimensional test problems. The median and interquartile range of DEI, PEI, and KB criteria are close. The median and interquartile range of DEI is larger. The number of cycles decreases with the updated point increasing. But the rate of decrease is slowing down, which indicates that the optimization rate cannot increase linearly as the number of update points increases. KB criterion has a lot of outliers when the updated point is great than two for the Branin function, which indicates that KB may be invalid when the number of the parallel updated points added is large. For the two-dimensional test function, the PMP criterion converges faster when the updated points are two. As the number of updated points increases, the number of iterations does not change much, indicating that the parallel infill sampling process does not accelerate. The MMP criterion has the problem of falling into the local optima. DMP criterion improves this problem and is better than the result of using the EI parallel criterion. The optimization efficiency of the DMP criterion is increased by 20% compared with the PEI criterion in the two updating points process. As the number of points increases, the efficiency improvement is no longer obvious The law of the parallel method including the PI criterion is similar to that of the EI criterion. But the optimization converges speed is low. The convergence value is scattered and a large number of abnormal points appear. For the three-dimensional test function, the medians of PEI, PPI, KB, and MPI methods all have a significant improvement, and the distribution of interquartile range is concentrated.

**Figure 8:**
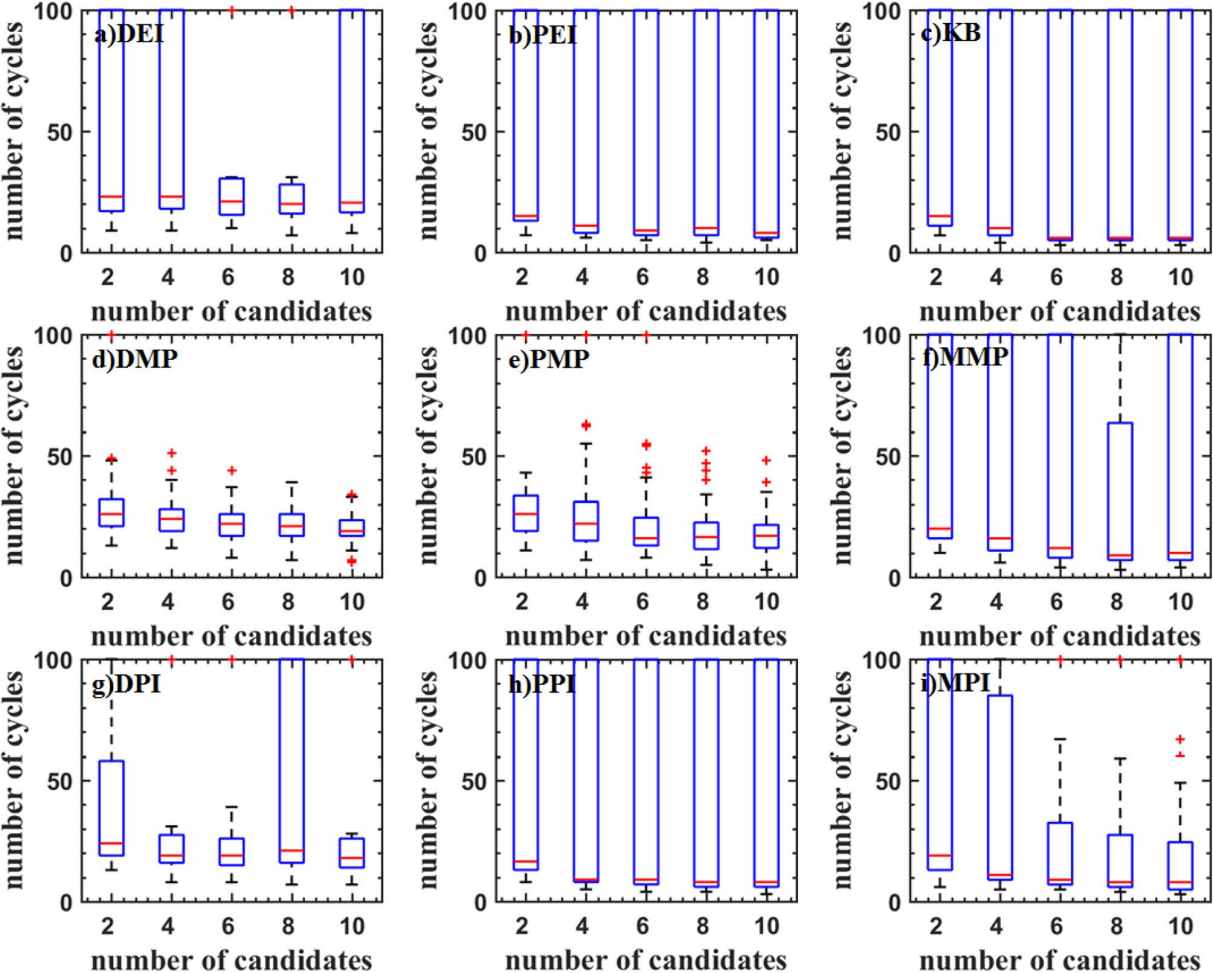
Test problems: Hartman6 function.

Figure 8 shows the test result of the Hartman6 function. Although the median is low, the interquartile range of DEI, PEI, KB, PPI, DPI, and MMP criteria are scattered. The main reason is that these criteria always converge near the optimal value. It can also be seen from the inverse design of airfoil. The DMP and PMP criteria have a high median and a concentrated interquartile range indicating that these criteria are more robust.

**Figure 9:**
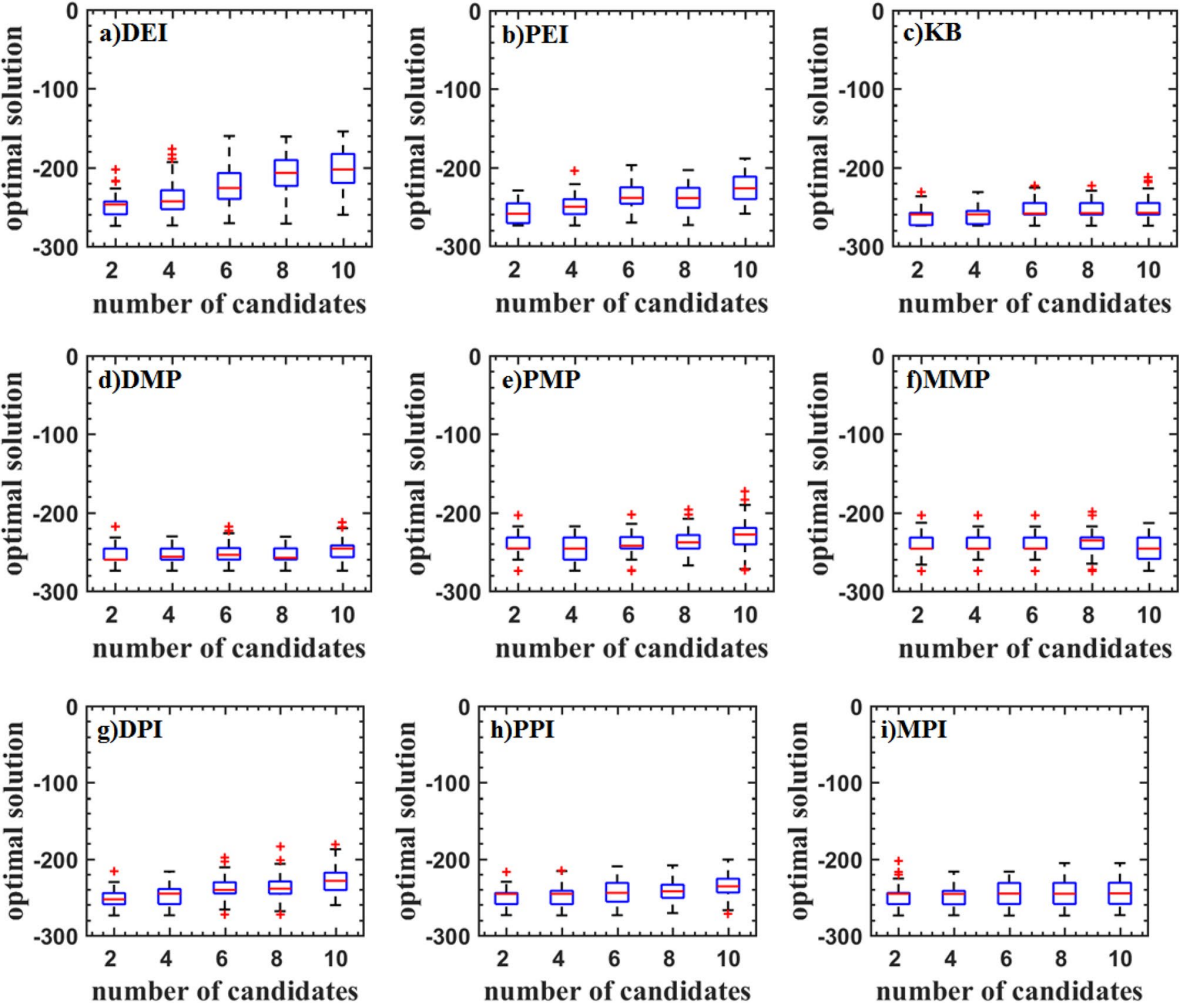
Test problems: StyblinskiTang7 function.

Figure 9 shows the test result of the StyblinskiTang7 function. With the increase of the number of updated points, the median of the optimal value is increased for DEI, PEI, DPI, MPI, PPI, and PMP criteria, which shows that the efficiency of parallel methods is reduced. The median of the optimal value of the KB, DMP, and MMP criteria is almost constant. The optimal values DMP criteria are low.

**Figure 10:**
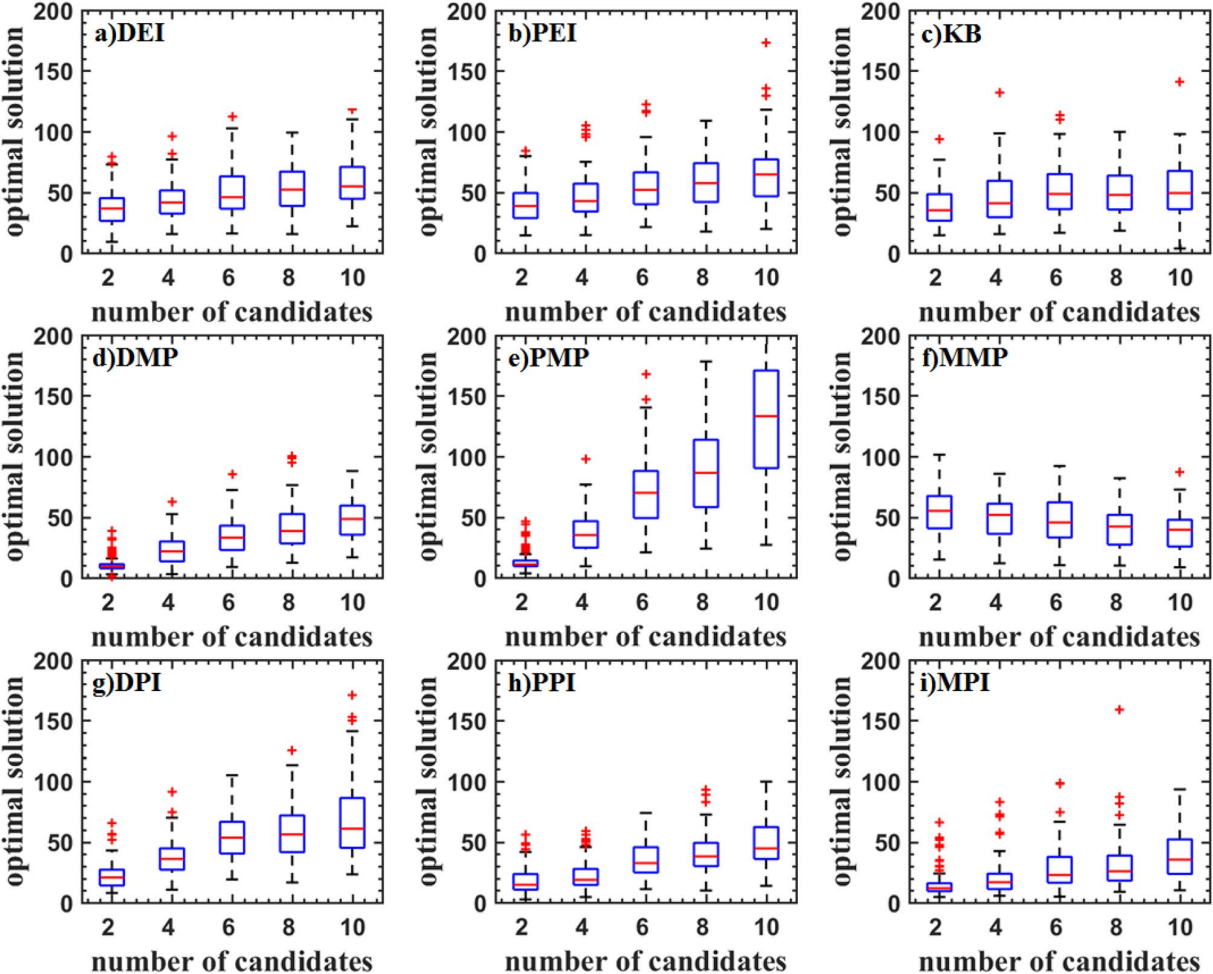
Test problems: Rosenbrock10 function.

The test result of the Rosenbrock10 function is shown in Fig. 10. When the updated points are two, DMP, PMP, DPI, MPI and PPI criteria have higher efficiency. As the updated points increase, the efficiency is gradually reduced. PMP criterion even traps in the local optimal solution. DEI, PEI, and KB criteria have a similar median. DMP, PPI, and MPI criteria have a smaller median.

**Figure 11:**
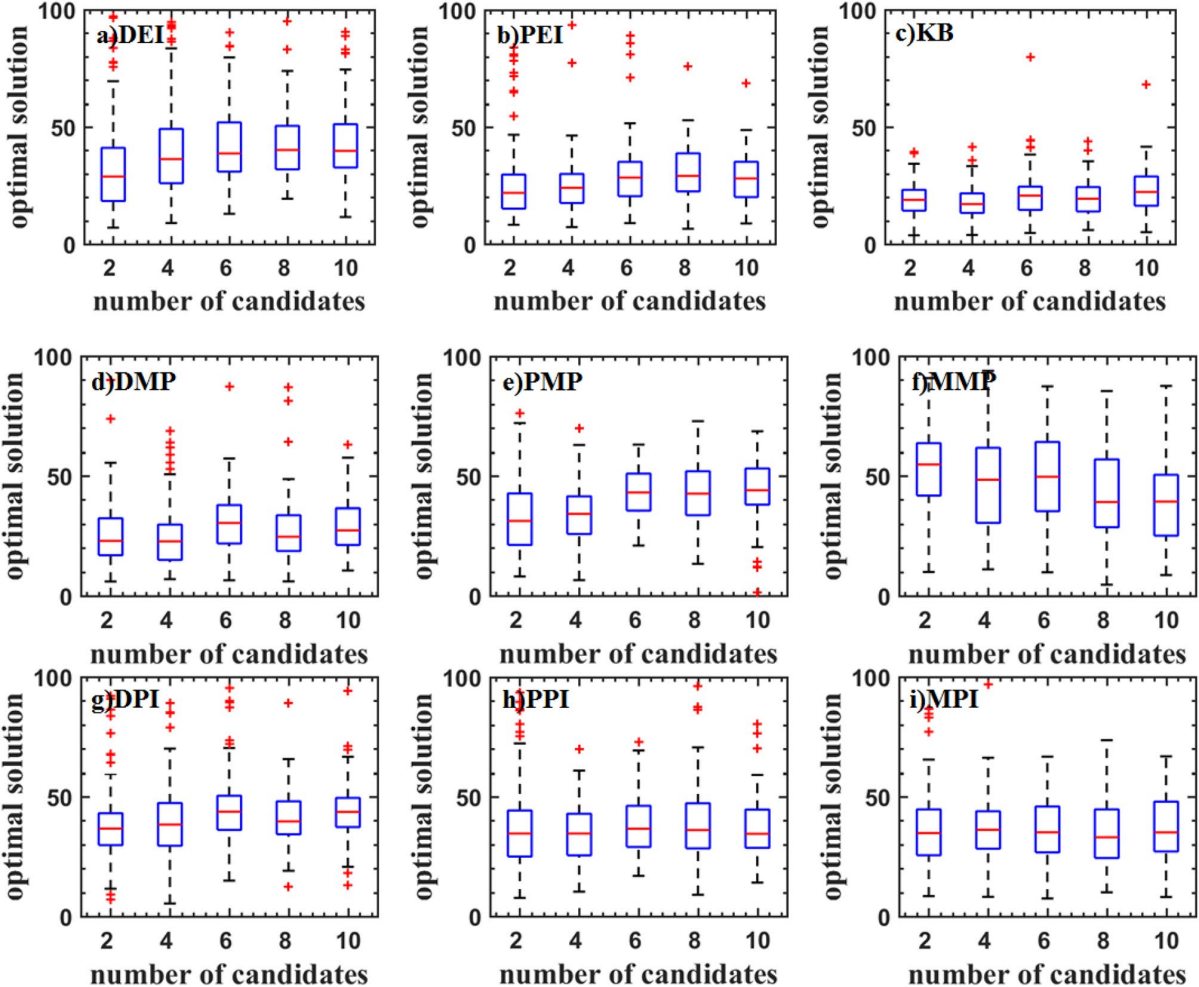
Test problems: Rastrigin12 function.

The test result of the Rastrigin12 function is shown in Fig. 11. It can be found that the optimal values obtained by DMP, PEI, and KB criteria are lower. And the interquartile range is narrower, but there are some outliers. The efficiency of parallel criteria for the high dimensional problems is decreased. KB criterion has a lower median and fewer outliers.

The results of test functions show that the adaptive distance function proposed in this paper effectively improves the problem that the traditional distance function is easy to fall into local optima. At the same time, after it is introduced into the MP criterion, its optimization rate is also optimal.

After the pseudo method is introduced into the MP criterion, although the problem of falling into the local optima is improved, the optimization rate is relatively slow. After the introduction of the PI criterion, the optimization rate is similar. The optimization rate of the DEI method for low-dimensional problems is slower, but it is not easy to fall into the local optima. The optimization rate of the DMP method is improved, and at the same time, it does not fall into the local optima.

## Application in the inverse design of airfoil

### Demonstrative example

The efficient global optimization (EGO) algorithm based on the Kriging model is widely used in airfoil design and optimization^21,22^. In this paper, the difference between the pressure distribution of the designed airfoil and the target pressure distribution is taken as the objective function. And the inverse design problem is transformed into the optimization problem.

21 $$Obj. = \sum\limits_{i = 1}^{N} {\left( {C_{p}^{i} - \hat{C}_{p}^{i} } \right)^{2} }$$

where the $C_{p}$ is the pressure coefficient of the target airfoil and $\hat{C}_{p}^{{}}$ is the pressure coefficient of the designed airfoil. $N$ is the number of points on the airfoil surface.

**Figure 12:**
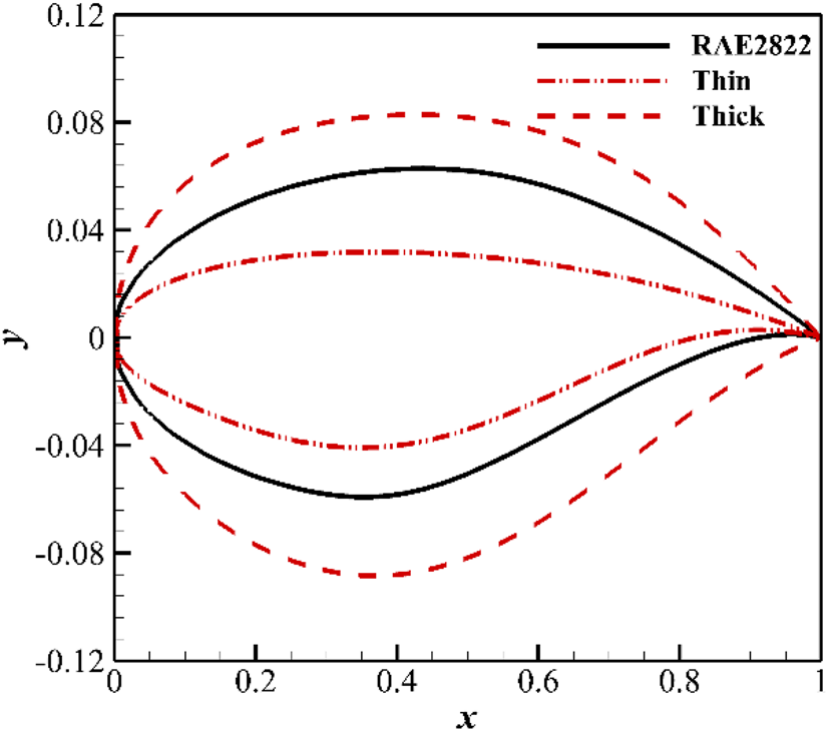
The design space for RAE2822 airfoil.

**Figure 13:**
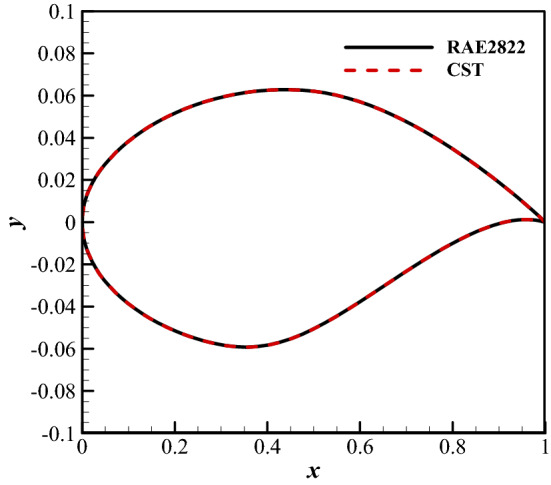
Comparison of the shapes of the designed airfoil and target airfoil.

The inverse design of the airfoil is performed using the CST method^23–25^. We first give a “thick” shape and a “thin” shape and then fit the two shapes by CST. We consider the CST parameters of the “thin” shape as the lower bound of the design variables and the parameters of the “thick” shape as the upper bound. The target airfoil should be included in the design space. The design space is shown in Fig. 12. The fourth-order CST method is used, and the total number of variables is 10. It can be seen from Fig. 13 that the RAE2822 airfoil can be reproduced.

### Numerical simulation method and verification

**Figure 14:**
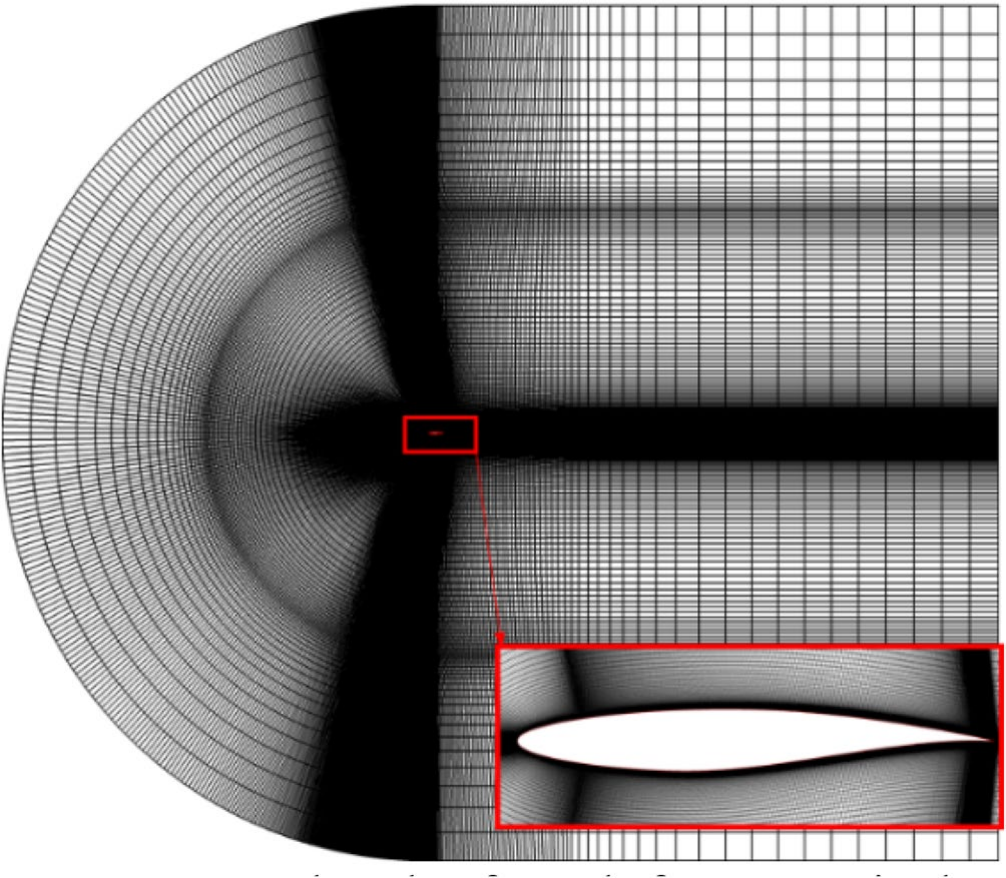
Sketch of mesh for numerical calculation.

**Figure 15:**
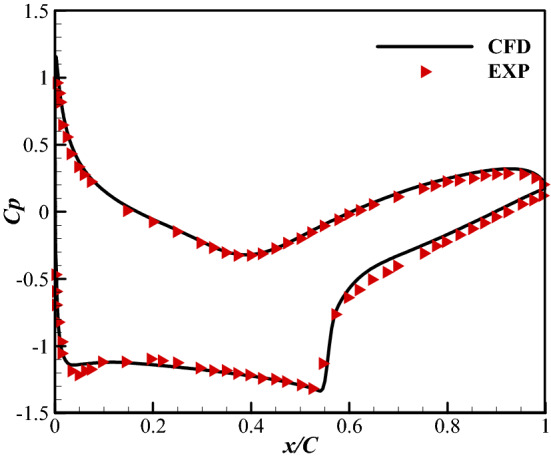
Comparison of CFD and experimental data.

The two-dimensional mesh is generated by Gambit. The computing domain shown in Fig. 14 is 70 m × 60 m, and the number of computing grids is about 220,000. The numerical simulation is performed using ANSYS Fluent 14.0. The turbulence model is SST k–ω. The inlet Mach number is 0.734, the inlet angle of attack is 2.79°. The Reynolds number is 6.5 × 10^6^. Figure 15 shows the numerical results and the experimental results of the pressure distribution on the airfoil surface. The numerical results are consistent with the experimental results. So the numerical method used in this paper has a high-level accuracy.

The initial samples are generated by LHS, and the total number of samples is 40. First, three single point criteria, EI, MP, and PI, are tested. The maximum number of cycles is set to 300. Later, parallel infill sampling criteria, KB, PEI, DMP, PMP, and MPI, were tested. The maximum number of cycles is set to 300, 200, 100 corresponding to adding 2, 3, and 6 updated points. A combination of the EI and MP method is also tested.

**Figure 16:**
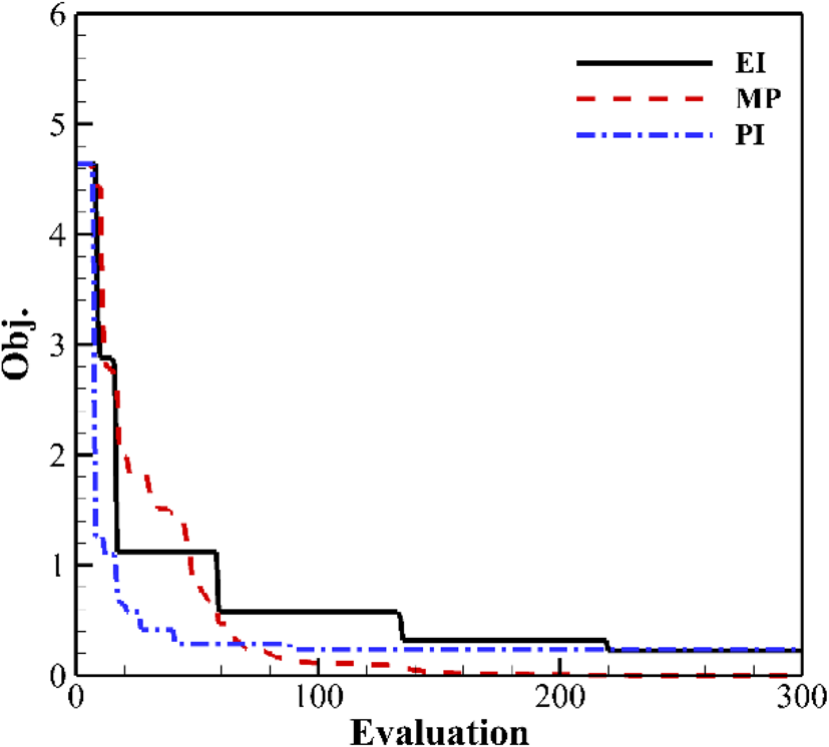
Convergence history of single updated point.

Figure 16 shows the result of the single updated point. The convergence curve of the MP criterion continues to decrease. It converges to 0 after 120 cycles. The convergence rate of MP in the early stage is slower than the others. The convergence rate of the PI is the fastest. EI and PI criteria only converge to about 0.3.

**Figure 17:**
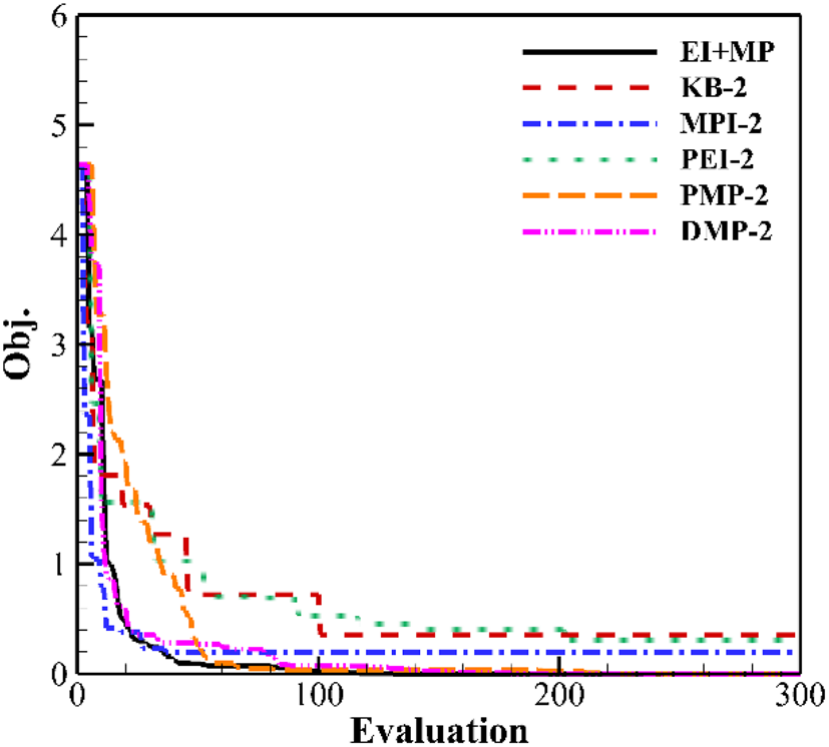
Convergence history of two updated points.

Figure 17 shows the result of two updated points. EI + MP, MPI, and DMP criteria have a higher convergence rate. The convergence rate of the PMP criterion is gradually slowed down, but it can also converge to zero in the end. The convergence rate and optimal value of the KB and PEI criteria are similar. The optimal value is about 0.3.

**Figure 18:**
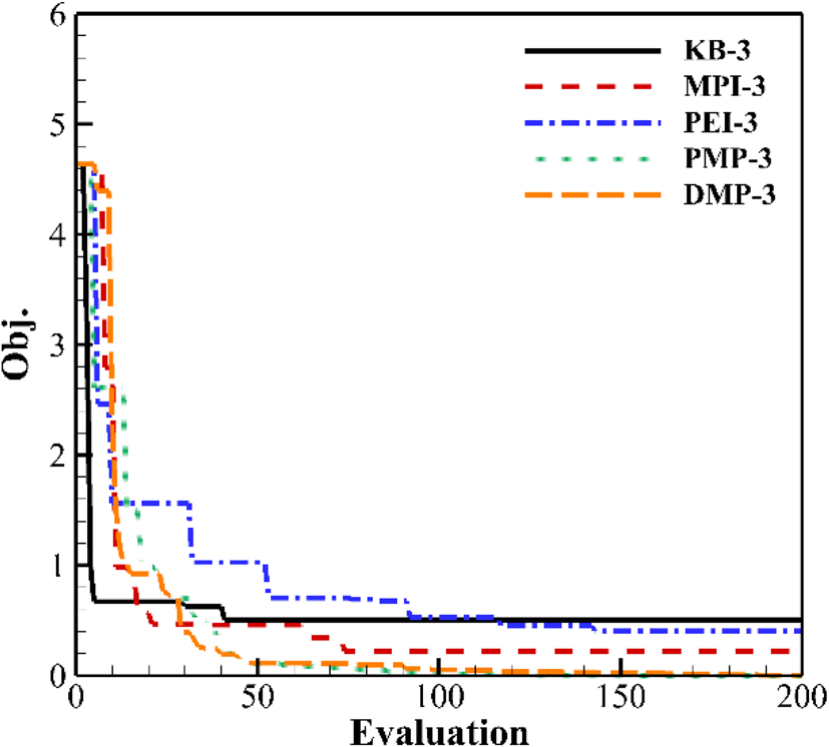
Convergence history of three updated points.

**Figure 19:**
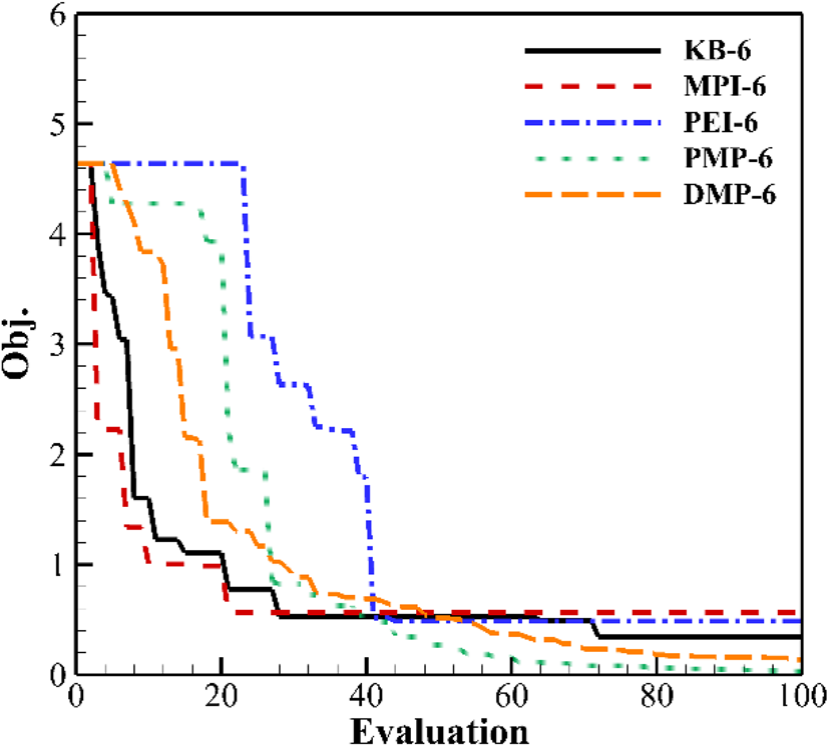
Convergence history of six updated points.

Figures 18 and 19 show the result of three and six updated points. KB has the fastest convergence rate, but only converges to about 0.4. As the number of updated points increases, the convergence rate of PMP and DMP criteria decreases. The optimal value still converges to 0. The convergence rate of PEI in the early stage decreases. The overall efficiency increases.

The results of the inverse design of the airfoil show that the DMP criterion also shows a better convergence rate and a smaller convergence value. The MPI method has a higher convergence rate in the early stage and the optimal value is higher. Therefore, the MPI criterion can be used in the early stage to accelerate the convergence speed in practical applications and later adopt the parallel method including MP criterion to obtain the optimal value.

## Conclusions

In this study, the “pseudo” method is applied to the MP criterion and PI criterion. An adaptive distance function is proposed. Test problems were used to evaluate these criteria to verify the effectiveness of these methods. Some parallel criteria are used in the inverse design of an airfoil to compare the differences between these methods in the optimization speed and the optimal value, which can provide a reference for actual use selection.

After the pseudo method is introduced into the MP criterion, although the problem of falling into the local optima is improved, the optimization rate is relatively slow. After the introduction of the PI criterion, the optimization rate is close to the PEI criterion. The optimization rate of the DEI method for low-dimensional problems is slower, but it is not easy to fall into the local optima. The optimization rate of the DMP method is improved, and at the same time, it does not fall into the local optima. Through the comparison of various parallel criteria, the DMP, DMP, PPI, PEI, and KB methods have better convergence speed and robustness.

The results of the inverse design of airfoil show that the parallel methods including the MP criterion have the best optimization value The convergence rate of the MPI is faster than the PEI and the KB, but the optimization values are close. Moreover, the MPI criterion can be used in the early stage to accelerate the convergence speed and later adopt the parallel method including the MP criterion to obtain the optimal value.

## Data Availability

The authors declare that the data supporting the findings of this study are available within the paper and additional data on methods used are available upon reasonable request.

## List of symbols

$C_{p}$: Pressure coefficient of the target airfoil
$N$: The number of samples points on the airfoil
n: Number of samples
$n_{\dim }$: Number of design variables
*q*: The number of updated point
$$R$$: Correlation function
$\hat{s}$: Mean squared error
$$S$$: Initial samples
$y$: Function values of initial samples
$$\hat{y}$$: Predicted value
$$z$$: Stochastic process
$\mu$: The mean of the Gaussian process
$\theta$: Parameters of the correlation function
$\delta$: Threshold value of distance function
$$\sigma^{2}$$: Process variance
$$\kappa$$: Improvement factor.
$$\Phi$$: Cumulative distribution
$\phi$: Probability density function

## Abbreviations

CST: Class-shape transformation
DEI: Parallel expected improvement criterion with the distance function
DMP: Parallel minimizing the prediction criterion with the distance function
DPI: Parallel probability of improvement with the distance function
EI: Expected improvement
EGO: Efficient global optimization
IF: Influence function
GA: Genetic algorithm
KB: Kriging Believer
LHS: Latin Hypercube Sampling
MP: Minimizing the prediction criterion
MMP: Parallel minimizing the prediction criterion with a fixed distance
MEI: Parallel expected improvement criterion with a fixed distance
MPI: Parallel probability of improvement with a fixed distance
P_EI_: Pseudo expected improvement criterion
PMP: seudo minimizing the prediction criterion
PI: Probability of improvement
PPI: Pseudo parallel probability of improvement
DIS: Distance function

## Subscripts

min: Minimum
max: Maximum
